# Assessment of Educational Needs and Quality of Life of Chronic Hepatitis Patients

**DOI:** 10.1186/s12913-017-2082-x

**Published:** 2017-02-17

**Authors:** Ming-Chuan Chen, Hung-Chang Hung, Hsiu-Ju Chang, Sheng-Shun Yang, Wen-Chen Tsai, Shu-Chuan Chang

**Affiliations:** 1 Department of Health Services Administration, China Medical University, No.91, Hsueh-Shih Road, 40402 Taichung, Taiwan; 2 Department of Healthcare Administration, Central Taiwan University of Science and Technology, No. 666, Buzih Road, Beitun District, Taichung, 40601 Taiwan; 3 Ministry of Health and Welfare Nantou Hospital, No. 478 Fuxing Rd., Nantou City, 540 Nantou County Taiwan; 4 Department of Nursing, Lee’s Medical Corporation, No. 2 Bade St., Taichung, 43748 Taiwan; 5 Division of Gastroenterology and Hepatology, Department of Internal Medicine, Taichung Veterans General Hospital, No. 1650, Taiwan Boulevard Sec. 4, 40705 Taichung, Taiwan; 6 Department of Nursing, Central Taiwan University of Science and Technology, No. 666, Buzih Road, Beitun District, Taichung City, 40601 Taiwan

**Keywords:** Chronic hepatitis, Educational needs assessment, Quality of life

## Abstract

**Background:**

Patient education is crucial in improving the health-related quality of life (HRQOL) of patients. At the same, understanding the concerns and needs of patients is essential in providing appropriate education. This study assessed the educational needs and HRQOL experienced by chronic hepatitis patients.

**Methods:**

We developed structured questionnaires with satisfactory validity and reliability to assess the educational needs of patients. HROQL was measured using a generic Short Form 36 (SF-36) and a liver disease-specific Chronic Liver Disease Questionnaire (CLDQ). Descriptive statistic measures and Pearson’s correlation analysis were applied for data analysis.

**Results:**

A total of 135 subjects were recruited from two regional teaching hospitals in Taiwan. “Disease characteristics and management” exhibited the highest mean score (3.17) among all the subscales of educational needs. In comparison with those without antiviral therapy, chronic hepatitis patients undergoing antiviral treatment scored significantly higher on all subscales of educational needs, especially on “side effects of antiviral treatment” (*p* < 0.010). The median range of the physical component summary score was 45.94, the mental component summary score was 49.37, and the mean CLDQ was 5.70. Several domains of educational needs were significantly inversely correlated with the CLDQ and SF-36 subscales.

**Conclusions:**

Education is highly required by chronic hepatitis patients, especially those receiving antiviral therapy and patients with poor HRQOL. These findings can serve as a useful reference for nursing personnel who perform needs assessment to develop individual nursing instruction and thereby improve the quality of care for chronic hepatitis patients.

## Background

Chronic hepatitis is a global issue that leads to a high rate of morbidity and mortality in humans [1]. The major etiologies of chronic hepatitis are viral infection and nonalcoholic fatty liver disease in most countries [2, 3]. As the disease progresses, patients infected with viral hepatitis, primarily hepatitis B and C, may develop cirrhosis or hepatocellular carcinoma (HCC). According to the World Health Organization report in 2015, nearly 240 million people are infected with the hepatitis B virus (HBV), and 130–150 million are infected with the hepatitis C virus (HCV) worldwide. Among these patients, approximately 65 million develop HCC [4]. In Taiwan, the prevalence rates of HBV and HCV are 17.3% and 4.4%, with an estimated 3 million HBV and 420,000 HCV carriers, respectively [5]. As a result of the complications of chronic liver inflammation or fibrosis, their negative effects on quality of life, particularly in terms of fatigue, financial burden, and depression, are immeasurable among chronic hepatitis B and C patients [6, 7].

Previous studies clearly demonstrated that patients with chronic B or C hepatitis are associated with a reduced health-related quality of life (HRQOL), as determined on the basis of the generic Short Form 36 (SF-36) or disease-specific questionnaires, such as the Chronic Liver Disease Questionnaire (CLDQ) [8–11]. The negative effects of such diseases on the quality of life of patients with or without antiviral therapies are profound, especially for chronic hepatitis C (CHC) patients who receive interferon (IFN)-based treatment [12–14]. Moreover, some CHC patients may be ineligible to receive IFN-based antiviral therapy because of psychiatric morbidities [15, 16].

The quality of life of chronic hepatitis patients could be improved by providing them with adequate nursing counseling and intensive education. Educational interventions are applied as a regular portion of the nursing work; it can be delivered in the form of designed activities, including symptoms management, lifestyle instructions, and behavioral modification for the benefit of chronic hepatitis patients [17]. Several studies have shown the positive effects of educational interventions on the HRQOL of chronic hepatitis patients [18, 19]. However, before drafting instructions, nurses must understand the educational needs of patients and the factors affecting those needs. A study on patients’ informational needs emphasizes that the program provided by nursing staff must meet the perspectives of patients clearly and clarifies unique priorities from the patients instead of the nurses [20]. Other studies have pointed out that the important information received by patients tends to differ according to the cognition of nursing staff [21, 22]. For example, Grogan (2010) reported that many HCV patients did not feel supported by nurses in terms of sleep management, which was a deeply concerning issue for the patients [23]. Another example is that members of the nursing staff misunderstand the needs of patients with regard to discharge information because they tend to decide on the educational needs of patients according to their own clinical experiences [24]. Under such circumstances, the patients’ actual health needs and concerns and their quality of life could be severely compromised. Moreover, they could experience anxiety and frustration from the disruption of their discharge plan. Therefore, the degree and type of curriculum content related to the patient concerns and the quality of life experienced by patients should be considered prior to the development of an appropriate informational support at different disease stages. Thus far, the educational needs of chronic hepatitis patients have not been adequately investigated. In the current work, we investigated the educational needs and HRQOL of chronic hepatitis patients by using the SF-36 and CLDQ subscales with the goal of answering the following questions. (1) What are the educational needs of chronic hepatitis patients? (2) What is the status of the HRQOL of chronic hepatitis patients?

## Methods

### Study design and sample

This work is a cross-sectional study. We used questionnaires to interview chronic hepatitis patients at outpatient clinics of two regional teaching hospitals in central Taiwan. The enrolled chronic hepatitis patients were those who tested positive for the HCV antibody or HBsAg for more than 6 months, exhibited an elevated serum alanine aminotransferase (ALT) level (above the upper limit of normal), and were diagnosed by experienced hepatologists. Patients with autoimmune hepatitis, alcoholic hepatitis, non-alcoholic steatohepatitis, cirrhosis of liver, and hepatocellular carcinoma were excluded.

### Ethical consideration

The processes of collecting and reviewing patients’ data and completing questionnaires were approved by the Institutional Review Board (IRB) of the investigators’ affiliated hospital (IRB reference number CCHIRB No: 121110).

### Measurements

#### Educational needs assessment

The educational needs questionnaire described the need of chronic hepatitis patients to understand diseases, treatment procedures, effects and side effects of drugs, management of side effects, diet restrictions, importance of each test, and daily care. The questionnaire comprised 15 items that were constructed by a native Chinese-speaking research nurse and six clinical case managers of chronic hepatitis. The questionnaire was completed and evaluated by five chronic hepatitis patients as a pilot test to evaluate comprehensibility. The questionnaire was validated by three experts from related fields of hepatology based on level of importance, clarity, and relevance of the content. The items were carefully selected, reformulated, or deleted, and an eight-item questionnaire was subsequently produced. The eight items that were included in the self-administered questionnaire were grouped into three domains on the basis of the judgment of the developers and feedback from the patients. The three domains were disease characteristics and management (three items), side effects of antiviral treatment (two items), and self-care programs (three items). Each item was presented in the form of a statement that described the need for instruction in the respective domain. The patients were asked to select one response from four choices (1 = *absolutely no need*, 2 = *a little need*, 3 = *mostly needed*, and 4 = *highly needed*; a high score reflected a high level of need) for each item on the basis their experience during the treatment period. The resulting instrument was evaluated using the content validity index (CVI) for validity and Cronbach’s alpha and test–retest reliability for reliability. The CVI value was 0.86 for each item, and the reliability coefficient of the two tests for the total scale was greater than 0.85–0.91.

#### HRQOL

The SF-36 consists of 36 items divided into eight scales, which are aggregated into two summary scores: a mental component summary (MCS) and a physical component summary (PCS). The eight scales are as follows: (a) physical functioning (PF), (b) role physical (RP), (c) bodily pain (BP), (d) general health (GH), (e) vitality (VT), (f) social functioning (SF), (g) role emotion (RE), and (e) mental health (MH). The scores for these eight SF-36 scales range between 0 and 100. High scores indicate excellent health. Regarding the Taiwanese version of the SF-36, the norms and internal consistency were validated by local researchers [25, 26]. The CLDQ, developed by Younossi et al. (1999), has been used in most recent studies on the HRQOL of chronic hepatitis patients. The questionnaire consists of 29 items divided into six domains: abdominal symptoms (AB), activity (AC), emotional function (EM), fatigue (FA), systemic symptoms (SY), and worry (WO). An overall CLDQ score was calculated for each domain, and the scores ranged from 1 (most impaired) to 7, which indicates the minimal frequency of symptoms; thus, a favorable HRQOL is presented. The total score was obtained as the average of the 29 items. The questionnaire exhibited satisfactory test–retest reliability and cross-sectional validity [27]. In 2011, the Traditional Chinese version of the questionnaire in Taiwan was translated and then published in recent studies [8, 28]. The reliability coefficient of the internal consistency for the total scale was 0.88–0.92.

#### Data collection

Each patient was interviewed during their outpatient clinic visit. The patients completed the questionnaires with the assistance of the interviewers. The baseline characteristics of the patients, namely, age, gender, marital status, educational level, current employment, monthly income, on-visit ALT levels, and treatment duration, were collected. The investigators administered three questionnaires, namely, the educational needs assessment, SF-36, and CLDQ.

#### Data analysis

A descriptive analysis was performed on the entire sample (*N* = 135) according to the baseline characteristics of the patients, their educational needs, the SF-36 questionnaire, and the CLDQ. Pearson’s correlation was used to examine the relationships among the educational needs assessment, CLDQ subscales, and SF-36 domains. All data analyses were conducted using SPSS software for Windows 14.0 (SPSS Inc., Chicago, IL, USA). A two-sided *p* < .050 was considered statistically significant.

## Results

### Baseline characteristics of chronic hepatitis patients

Among the 135 enrolled patients, 66 (48.9%) were male. Their ages ranged from 20 years old to 86 years old, with an average of 55.54 years old. More than half (51.1%) of the patients had a below high school education level. A total of 108 patients (80.0%) were married and lived with their families. Most of the patients (51.9%) were unemployed. The mean ALT level was 47.89 (SD = 57.21), and 88 patients (65.2%) were diagnosed with hepatitis C. Exactly 85 of the patients (63.0%) were receiving antiviral therapy during the study visits (Table 1).

**Table 1:** Demographic and baseline characteristics of study patients (n = 135)

Variables	Categories	n	%
Gender	Male	66	48.9
	Female	69	51.1
Marital status	Married	108	80.0
	Single, divorce or widowed	27	20.0
Educational level	Elementary school and below	69	51.1
	Junior and Senior high school	38	28.2
	College and above	28	20.7
Occupation	Employed	65	48.1
	Unemployed	70	51.9
Histological diagnosis	Hepatitis B	47 88	34.8 65.2
	Hepatitis C		
Antiviral treatment	Yes	50 85	37.0 63.0
	No		
Age Mean (SD)	55.54 (15.72)		
ALT level^a^ Mean (SD)	47.89 (57.21)		

^a^ *ALT* alanine transaminase, *SD* standard deviation

### Educational needs assessment for chronic hepatitis patients

The average score of patients’ educational needs was 3.13 (SD = 0.78), with the highest mean score (3.17, SD = 0.80) assigned to “disease characteristics and management.” Among the eight items of educational needs, “treatment regimens and related procedures” received the highest score of 3.20 (SD = 0.82), and “relaxation skills on emotional stress or anxiety” received the lowest score of 3.03 (SD = 0.85). This result indicates that the patients were highly needed information on hepatitis therapy. The chronic hepatitis patients undergoing antiviral treatment also scored significantly higher on the three domains in comparison with those without antiviral therapy (*p* < 0.010), with the greatest score difference of 0.64 being on the domain of “side effects of antiviral treatment,” and the highest score is 3.54 (SD = 0.67). Among the eight items, “notifications on nutrition and diet” received the highest score (3.00) from the patients without antiviral therapy, whereas “treatment regimens and related procedures” received the highest score (3.57) from the patients with antiviral therapy (Table 2).

**Table 2:** The educational needs of chronic hepatitis patients

Variables	Total (n = 135)	No antiviral-treated chronic hepatitis patients (n = 85)	Antiviral-treated chronic hepatitis patients (n = 50)	*P* value
	Mean (SD)	Mean (SD)	Mean (SD)	
Disease characteristics & management	3.17 (0.80)	2.95(0.82)	3.53(0.62)	0. 001
Disease characteristics and diagnostic tests for chronic hepatitis	3.19 (0.81)	2.96 (0.84)	3.56(0.61)	0. 001
Transmission route and preventive strategies	3.13 (0.83)	2.92(0.85)	3.48(0.68)	0. 001
Treatment regimens and related procedures	3.20 (0.82)	2.96(0.84)	3.57(0.64)	0. 001
				0. 001
Side effects of antiviral treatment	3.14 (0.82)	2.90(0.82)	3.54(0.67)	0. 001
Adverse events (AE) due to treatment	3.16 (0.83)	2.93(0.84)	3.55(0.68)	0. 001
Strategies to improve AE	3.11 (0.84)	2.87(0.85)	3.53(0.68)	0. 001
Self-care strategies	3.10 (0.81)	2.94(0.83)	3.37(0.69)	0.002
Lifestyle maintenance and caring of daily activities	3.11 (0.84)	2.93(0.87)	3.40(0.70)	0.001
Notifications on nutrition and diet	3.15 (0.81)	3.00(0.83)	3.42(0.70)	0.003
Relaxation skills on emotional stress or anxiety	3.03 (0.85)	2.88(0.87)	3.28(0.76)	0.006

### Quality of life of chronic hepatitis patients

The total quality of life score was in the medium range. Table 3 shows the mean SF-36 score (69.77, SD = 21.47). Six domains of SF-36 yielded a mean score higher than 60, and two domains yielded a mean score lower than 60. Among the eight subscales of SF-36, “general health” received the lowest average score of 45.15 (SD = 26.14), and “social functioning” received the highest average score of 82.56 (SD = 26.55). This study disclosed average scores of 45.94 (SD = 14.55) and 49.37 (SD =13.75) for the PCS and MCS, respectively_._ The mean CLDQ (5.70, SD = 1.32) score was moderate; the highest score was for “worry” (6.15, SD = 1.14), and the two lowest scores were for “fatigue” (5.08, SD = 1.59) and “activity” (5.51, SD = 1.46), thereby indicating that most patients had experienced severe fatigue.

**Table 3:** Scores for quality of life in chronic hepatitis patients

Variables	Minimal value	Maximal value	Mean (SD)
Physical functioning	0	100	77.74 (27.77)
Role physical	0	100	68.70 (45.17)
Bodily pain	12	100	81.45 (23.57)
General health	0	97	45.15 (26.14)
Vitality	0	100	56.41 (26.29)
Social functioning	0	100	82.56 (26.55)
Role emotional	0	100	78.53 (39.79)
Mental health	4	100	67.64 (20.37)
PCS	12.2	70.7	45.94 (14.55)
MCS	10.6	82.2	49.37 (13.75)
Average SF-36 score	15	98	69.77 (21.47)
Abdominal symptom	1.3	7.0	5.98 (1.32)
Fatigue	1.0	7.0	5.08 (1.59)
Systemic symptoms	3.2	7.0	5.84 (1.01)
Activity	1.3	7.0	5.51 (1.46)
Emotional function	1.5	7.0	5.66 (1.30)
Worry	1.6	7.0	6.15 (1.14)
Average CLDQ score	2.4	7.0	5.70 (1.32)

### Relationship of educational needs with the SF-36 and CLDQ scales

The relationship between educational needs and the SF-36 and CLDQ subscales is presented in Table 4. Almost all domains of educational needs exhibited a negative significant correlation with the SF-36 and CLDQ subscales (*r* > −0.18). In particular, the lower scores of VT from SF-36 and AT from the CLDQ were significantly inversely correlated (*r* > −0.20) with the three domains of educational needs. Only “self-care strategies” showed a weak negative association with the CLDQ subscales.

**Table 4:** Pearson’s correlation coefficients between the educational needs with SF-36, CLDQ

Variables	The need of patient education		
HRQOL	Disease characteristics & management	Side effects of antiviral treatment	Self-care strategies
PF	-.14	-.15	-.12
RP	-.37**	-.34**	-.33**
BP	-.39**	-.39**	-.32**
GH	-.13	-.12	-.08
VT	-.27**	-.28**	-.21*
SF	-.25**	-.26**	-.23**
RE	-.27**	-.25**	-.24**
MH	-.23**	-.23**	-.15
Average SF-36 score	-.36**	-.36**	-.31**
AB	-.21*	-.18*	-.10
FA	-.18*	-.20*	-.15
SY	-.21*	-.23**	-.14
AT	-.25**	-.27**	-.20*
EM	-.15	-.16	-.05
WO	-.15	-.18*	-.07
Average CLDQ score	-.26**	-.28**	-.17

*p < .05.**p < .01

## Discussion

This questionnaire presented in this work is the first reported educational needs assessment questionnaire for chronic hepatitis patients. The key results of this study demonstrated that the chronic hepatitis patients exhibited a strong desire for patient education, particularly for information on “disease characteristics and management.” The CLDQ mean score of 5.70 obtained in this study indicated a moderate decrease in the quality of life of the chronic hepatitis patients. Additionally, the SF-36 mean score of 69.77 revealed that the experiences of the participants were not well in general. Our data also revealed that all of the domains of educational needs were significantly associated with most subscales of SF-36, except PF and GH. In addition, we noticed that the “disease characteristics and management” and “side effects of antiviral treatment” subscales of educational needs were significantly negatively correlated with CLDQ. This finding hinted that patients with decreased scores of SF-36 and CLDQ needed additional education programs for disease treatment. In particular, the CHC patients receiving antiviral treatment required additional information on “side effects of antiviral treatment.”

### Educational needs for chronic hepatitis patients with or without antiviral treatment

Pondering on the various HRQOL experiences that classify the educational needs of chronic hepatitis patients is an essential step for nursing staff to develop appropriate educational programs. However, no study has determined whether a considerable need for patient education exists or whether such need is connected with HRQOL. In this study, all chronic hepatitis patients ranked “disease characteristics and management,” especially “treatment regimens and related procedures,” as the most critical educational need. In comparison with those without antiviral therapy, the chronic hepatitis patients undergoing antiviral treatment scored significantly higher on all items, especially on “strategies to improve AE,” which had a maximal reduction score of 0.66. Hence, the information on the “side effects of antiviral treatment” must be emphasized among patients receiving antiviral therapy. Similar to the findings of a previous study [29], the hepatitis C patients in the current work rated the importance of HCV health care needs, particularly those related to “modes of transmission and treatment” and “the quality HCV medical care.” Although “self-care strategies” received the lowest scores, most of the chronic hepatitis patients, especially the CHC patients without antiviral treatment, reported that they were eager to receive information on “notifications on nutrition and diet.” Nutritional therapy improved the quality of life of CHC patients [30]. Therefore, the informational needs of participants in the area of “notifications on nutrition and diet” must be thoroughly addressed in patients’ educational courses. Developing educational and supportive programs that meet these educational needs assessment may be useful as a key task in the nursing service [31]. Moreover, the minimal educational need for “relaxation skills on emotional stress or anxiety” is likely a result of cultural influence because Taiwanese people are typically conservative with regard to emotional expression; this tendency prevents them from proactively asking for help. This finding is consistent with that of a qualitative study, in which the Chinese patients did not share their emotional and social needs with nurses [32]. However, the mental health and social needs of patients are significant parts of life [33, 34], and this situation must be addressed in the development of teaching strategies and curricula. Nurses in Taiwan should be aware of the psychosocial needs of patients and be able to proactively advice chronic hepatitis patients about relaxation skills to reduce emotional stress or anxiety.

### Relationship between educational needs and HRQOL

Three domains of patient educational needs were significantly correlated with SF-36 and the CLDQ (*r* > 0.18), with the correlation ranging from weak to moderate. Almost all the SF-36 subscales were significantly correlated with the three domains of educational needs, with the greatest negative correlation coefficients (*r* = −0.39) being between the “disease characteristics & management” and “side effects of antiviral treatment” subscales and BP of SF-36. Hence, the patients who exhibited a high score in BP (less bodily pain) did not require additional instructions, whereas most of the patients required further instruction to improve their HRQOL. This result can help healthcare personnel to understand the increased requirement for educational needs from poor HRQOL patients.

### Study limitation and suggestions

In this study, we identified the educational needs of chronic hepatitis patients that should be considered in the future development of intervention programs on patient education. The results may serve as reference for nurses to ensure an excellent management of patients’ discomfort and their self-care ability. Patient education programs must focus on the individual needs of patients and the continuous assessment of such needs [23, 35]. Furthermore, we suggest that updated counseling and materials regarding self-care strategies must be included in the curriculum, particularly in the area of nutrition. This study has its limitations because all the subjects were recruited from two regional teaching hospitals in Taiwan and this study was designed mainly for patients with chronic hepatitis B and C. Thus, the results may not be applied to other chronic hepatitis patients.

## Conclusions

In this study, we delineated a fair HRQOL of chronic hepatitis patients in Taiwan. In addition, we highlighted the importance of patient education assessment based on the perspectives and needs of patients. We suggest that counseling and educational materials should be updated, and an appropriate nursing management scheme needs to be designed for chronic hepatitis patients.

## Abbreviations

ALT: Alanine aminotransferase
CHC: Chronic hepatitis C
CLDQ: Chronic liver disease questionnaire
HBV: Hepatitis B virus
HCC: Hepatocellular carcinoma
HCV: Hepatitis C virus
HRQOL: Health-related quality of life
IFN: Interferon
MCS: Mental component summary
PCS: Physical component summary
SF-36: Short form 36
